# Medical Items

**Published:** 1908-01

**Authors:** 


### MEDICAL ITEMS.

While LA GRIPPE is an acute infectious disease, it is more than this. The rapidly multiplying germs evolve a toxin which adds to the original infection a state of toxaemia. It is for this reason that Alkalithia, as an ideal eliminant, is of such service as a supplemental treatment, and when given in teaspoonful full doses, every four hours, will modify the severity, and shorten the duration of this disease.

---

### NASAL CATARRH.

BY C. W. CANAN, A. B., M. D., ORKNEY SPRINGS, VA.

Chronic nasal catarrh is, for convenience, divided into three stages or varieties: (1) Simple Rhinitis, (2) Hypertrophic Rhinitis, (3) Atrophic Rhinitis. In the first form there is a simple chronic inflammation of the pituitary membrane, uncomplicated by serious structural changes, but attended with a discharge of mucous or mucopus. When this condition is allowed to continue for any considerable length of time, structural changes take place in the mucous membrane and we have Hypertrophic Rhinitis as the result. There is usually an excessive secretion of thick tenacious mucus, which more or less occludes the nasal passage. Enlargement of the adenoid elements in the adjacent parts of the pharynx coexist as a rule. In this form of the disease the inferior turbinated body suffers the most frequently and to the greatest extent; this in turn may irritate the neighboring pharyngeal parts, leading to Chronic Pharyngitis, catarrh of the eustachian tube and even Chronic Laryngitis. The third form of the disease, or Atrophic Rhinitis follows as a sequence of the former or second variety. It is the result of contraction of fibrous tissue formed upon and around the vessels and glandular structures and consequently their obliteration. The membranes are dry and covered with dry scabs and muco-pus which become more offensive and abundant as the disease progresses. The olfactory sense may be partially or wholly lost. We give this brief history of pathology to prove that a case of simple nasal catarrh may turn out to be a grave disease in the end if not promptly recognized and properly treated. "Clean up and keep clean" should be our motto. The remedy best suited for this purpose should be one that is alkaline in reaction, non-irritating and contain enough antiseptic qualities to disinfect the nasal cavity without producing toxic symptoms. The remedies that have been in general use are Carbolic Acid, Acid Salicylic, Acid Boracic and Sodii Bicarb. in solution or in combination, but we have found a remedy that surpasses these as well as others in results. This is Glyco-Thymoline. This remedy can be used in all percent. solutions up to the plain drug according to the sensitiveness of the nasal mucous membrane and the condition for which it is prescribed. In the treatment it is necessary to remove all pus, mucus, scabs, scales and dead bone if any be present. To remove all these except the last, Glyco-Thymoline should be used through a syringe until the entire tract is clean, then if there be any specially diseased points it should be applied to them in full strength. In all mild cases of catarrh a thorough cleansing with this remedy and keeping the cavity scrupulously clean afterwards with the same will be all that is necessary to effect a cure. That the reader may better understand the value of this remedy in this disease, we will report the clinical history of a few cases selected from our notes:

Case 1. Mr. F. M., farmer, age 37. Presented the following history: At times the nasal secretions were profuse and mixed with pus, at others the nose was very dry and breathing was difficult. Examination revealed a highly sensitive mucous membrane thickened and congested. The mucous follicles on the post pharyngeal walls were also enlarged and secreted a tough tenacious mucous which kept the patient hawking to clear his throat. Diagnosis: Chronic Rhinitis. Treatment consisted in cleansing the canal thoroughly with a twenty percent. solution of Glyco-Thymoline. The patient was then instructed how to douche the nose night and morning with the above solution of Glyco-Thymoline. The patient was then instructed how to douche the nose night and morning with the above solution and return once a week to my office. Improvement was rapid and at the end of one month patient was dismissed.

Case 2. Miss B. M., housekeeper, age 22. Good family history. Last March she contracted La Grippe in a severe form, following which more or less nasal trouble was in evidence. There were periods of sneezing, dryness and a decided flow of clear liquid. At times the dryness was so marked that she could not breathe through her nose. Finally the watery discharge became bloody and at times contained pus. Headache developed, head noises, dropping into throat and many other symptoms. Examination showed a highly congested pituitary membrane, while the anterior cavity was filled with dead scabs and scales. The lining to the eustachian tubes was so swollen that it was impossible for air to reach the middle ear. Diagnosis: Chronic Rhinitis. Treatment consisted in thoroughly cleansing cavity with a twenty percent. solution of Glyco-Thymoline. When all scabs were removed, the pituitary bodies were touched with the drug in full strength; the bag was then used to inflate middle ear and the nose and throat sprayed with the atomizer. Patient was instucted how to cleanse nasal cavity with the remedy three times daily and to come to the office twice a week. The improvement was decided from the time of the first treatment and in six weeks there was not a trace of the disease.

Case 3. Miss Lizzie R., age 16 years. She was very much reduced in flesh and was very anemic, complained of attacks of sneezing in which she would sneeze from eight to twenty times before she could stop. Nose would discharge freely, then become very dry. She also suffered headache, giddiness, and a continuous full feeling between the eyes. There was dropping into the throat at times and she had a troublesome cough with wheezing in chest. The mucous membrane was highly congested, covered in places with scabs and tough mucous that was difficult to dislodge. A deep angry ulcer presented on the anterior surface. There was also a Pharyngitis and Laryngitis and catarrh of the eustachian tubes. Diagnosis: Chronic Rhinitis complicated with Pharyngitis and Laryngitis. Treatment was same as in second case, except throat and larynx were sprayed daily with Glyco-Thymoline. Patient was also placed upon Fowler's Solution mv three times daily after meals and Cod Liver Oil one hour after meals. The condition showed marked improvement after the second week and at the end of another week patient was entirely well.

---

### ERYSIPELAS — PNEUMONIA.

---

W. E. SROFE, M. D., MARTINSVILLE, OHIO.

---

June 5, 1905, I was called to attend Mr. K—. I found him suffering with a very aggraveted case of facial erysipelas. I applied my usual treatment of carbolized salve locally, and gave the proper internal treatment, but when I saw the case again in twenty four hours, I found symptoms no better. I thought I would try Antiphlogistine. After applying the salve to face, I spread the Antiphlogistine on a cloth making a mask that would cover the entire face, directing nurse to change it when it dried out.

Next day I found tht patient much improved. He said "that clay relieved all the burning five minutes after you applied it." I now make it a rule to use Antiphlogistine in treating erysipelas, and I am sure my patients get along faster than they did than when treated without it.

I also use Antiphlogistine in pneumonia, and all cases of inflammation of the lung or pleura. Indeed, I would hate to have to treat this kind of cases without Antiphlogistine. I will report on one case of an infant, where I believe this remedy saved the patient's life.

Jan. 3, 1906, Infant, Age 18 months. Two days after initial fever, temp. 104 degrees, resp. 48, pulse 120; tongue coated, could hardly get breath, expiratory moans, crepitent rales. Gave internal treatment, and covered both back and front of chest with Antiphlogistine. In twenty-four houre the breathing was much better and temperature lower. On my third visit, I found all the symptoms so improved that I dismissed the case.

THE BEST RE-CONSTRUCTIVE

PHILLIPS'

PHOSPHO-MURIATE OF QUININE

Registered in the U. S. Patent Office, Oct. 17th, 1905.

(olSuePblhosphates wlth Muriate of Quinine, Iron and Strychnia)

THe Chas. H. Phillips Chemical Co. New York and London.

### LIEBERKUHN.

BY L. K. BALDAUF.

JOHN NATHANEAL LIEBERKUHN was born in Berlin, September 5, 1711. His father having decided in favor of the ministry, for three years, first in Halle, later in Jena, he applied himself to theological studies. Attracted, however, by the mathematical-physical lectures of Hamburger, he took up the study of natural sciences. His interest in this study was later increased through contact with Wedel and Lechmeyer who, together with Hamburger, fostered his scientific zeal and encouraged him to study medicine. In the year 1733, however, his father persisting, he betook himself to Roystock to further prepare himself for entrance into the ministry. Very soon after, before his ordination, his father died. Freed from all constraint, he determined to devote himself entirely to the natural sciences. After an extensive journey through Germany he went to Leyden, where he studied under Boerhaave, Albinus and Gaut, and in 1739, after his inaugural dissertation, "De valvula coli et usu processus vermicularis," received his doctor's degree. Later he visited London, where, after the presentation of anatomical specimens, he was elected a member of the Royal Society. After a short stay in practicing physician. He was made a member of the medical faculty. In October, 1756, he died.
Paris he returnd to his native city and established himself as a

Lieberkuhn combined with an uncommon gift of observation a remarkable technical ability. This enabled him to perfect for his anatomical studies certain necessary instruments, for instance his anatomical microscope. With the exception of his inaugural dissertation and the description of the microscope used by him for anatomical study, and a method for the histological study of the intestines, which appeared in the proceedings of the Berlin Academy of Science, he published only the excellent article concerning the intestinal villi (De fabrica actione villorum intestinorum teruim, 1745).

In this article, Lieberkuhn described for the first time the glandular structures which have since borne his name. He is no less renowned for his beautiful preparations of injected blood vessels. The four hundred specimens which, after his death, came into the possession of Biereis, and which were later distributed among various museums, are still in an excellent state of preservation.

REFERENCES.

PORTAL. *Histoire de l'Anatomie et da La Chirurgie,* T.V. 158.
*Biographisches Lexikon,* Berlin 1901, 1006.
*Biographisches Lexikon der hervorrageden,* Arzte, III, 704.

Albany Medical Annals, Oct. 1907.

### MISS FANNY ROBERTS APPOINTED LIBRARIAN FOR ELI LILLY & CO.

Eli Lilly & Company have established a library and reading room in connection with the scientific department and installed as librarian Miss Fanny O. Roberts, a graduate of the Purdue School of Pharmacy '06. In addition to her pharmaceutical training, Miss Roberts has a good general scientific education which equips her to assume charge of a technical library. Until recently, the reference books and periodical literature owned by the house, was scattered through the various departments, but for some time the growth of the scientific staff has emphasized the need of a central library organized along modern lines. The library is well supplied with the best works on chemistry, botany, physics, pharmacy, physiology, medicine and other subjects having a bearing on manufacturing pharmacy, in addition it receives the best periodical literature on these subjects published in English, German and French. Miss Roberts will give her entire time to its interests.

---

Boston, December 27, 1907.

Editor, Atlanta Journal-Record of Medicine:—

Will you kindly insert the following in your journal, giving it as prominent a place as possible?

The writer desires information regarding any alleged recoveries or cures of inoperable or recurrent carcinoma of the mammary gland.

If any case or cases are known to anyone who reads this circular and can be authenticated by facts as to the history and condition prior to recovery and the length of time which has elapsed since recovery, such information will be much appreciated and duly acknowledged.

Any well authenticated reports of recoveries from carcinoma located in other parts than the mammary gland will be welcomed.

Cancer paste cures, X-ray cures, radium cures, or cures as a result of surgical operation are not wanted.

**Hearsay cases are not wanted unless accompanied** by name and address of the person who can give knowledge first hand.

Address, Horace Packard,
470 Commonwealth Ave.,
Boston, Mass.

